# CLIC1 and CLIC4 demonstrate cell protective antioxidant activity against UV exposure

**DOI:** 10.3389/fcell.2025.1674374

**Published:** 2025-11-18

**Authors:** Khondker Rufaka Hossain, Amani Alghalayini, Daniel R. Turkewitz, Stella M. Valenzuela

**Affiliations:** School of Life Sciences, University of Technology Sydney, Sydney, NSW, Australia

**Keywords:** chloride intracellular ion channel (CLIC) proteins, CLIC1, CLIC4, fibroblast cells, keratinocyte cells, antioxidant, oxidoreductase activity, UV irradiation

## Abstract

**Background:**

Redox homeostasis is critical for maintaining healthy biological systems. Under physiological conditions, the human antioxidant defence system relies on enzymes such as superoxide dismutase (SOD), catalase (CAT), and glutathione peroxidase (GPx). Recent studies have shown that members of the Chloride Intracellular Ion Channel (CLIC) protein family, particularly CLIC1 and CLIC4, also exhibit antioxidant and cytoprotective activities. Overexpression of these proteins confers cellular protection, whereas their knockdown increases susceptibility to oxidative stress.

**Methods:**

This *in vitro* study investigated the antioxidant and cellular protective effects of CLIC1 and CLIC4 against UV-induced damage in human skin cells. Comparative analyses were performed using known endogenous antioxidant proteins, glutaredoxin (Grx) and Glutathione S-transferase-Omega (GST-Ω), as well as the antioxidant drug N-acetylcysteine (NAC). Recombinant purified CLIC proteins (rCLIC1 and rCLIC4) were added exogenously to skin cells, while CLIC knockdown models were used to assess loss-of-function effects.

**Results and Discussion:**

Exogenous addition of rCLIC1 and rCLIC4 provided significant cellular protection against UV-induced oxidative damage, reducing reactive oxygen species (ROS) production. In contrast, knockdown of CLIC1 or CLIC4 increased cellular vulnerability to oxidative stress. The protective and antioxidant activities of rCLIC1 and rCLIC4 were comparable to those of Grx, GST-Ω, and NAC. These findings highlight the potent antioxidant and cytoprotective roles of CLIC1 and CLIC4 in maintaining cellular redox balance. The ability of exogenously added recombinant CLIC proteins to mitigate oxidative and UV-induced damage suggests potential therapeutic applications for this protein family in oxidative stress-related conditions.

## 1 Introduction

Chloride Intracellular Ion Channel Proteins (CLIC) are a family of six proteins, CLIC1-6 in vertebrates (Argenzio and Moolenaar, 2016; Cromer et al., 2002; Gururaja et al., 2018; Littler et al., 2010) that are also evolutionary conserved across species (Littler et al., 2008), with CLIC-like proteins identifed in plants (Elter et al., 2007), bacteria (Gururaja et al., 2017), insects and worms (Littler et al., 2008). The CLICs have been studied for their interactions with cell membranes (Goodchild et al., 2009; Hossain et al., 2019; Hossain et al., 2023; Littler et al., 2004; Singh and Ashley, 2006; Tulk et al., 2002), along with their ion channel activity (Tulk et al., 2002; Al Khamici et al., 2016; Averaimo et al., 2013; Tulk et al., 2000; Valenzuela et al., 2013) and more recently their glutathione-dependant oxidoreductase enzymatic functions (Al Khamici et al., 2015; Alghalayini et al., 2023; Hernandez-Fernaud et al., 2017; Turkewitz et al., 2021). This latter enzymatic function is less well characterised and has most recently been shown by us (Hossain et al., 2025) and others (Al Khamici et al., 2022), to afford antioxidant protection to cells undergoing oxidative stress. Controlling cell oxidative stress is key for cell health and much focus has been given to developing agents that can alleviate and protect cells. Though widely used, small molecule free radical scavengers are inefficient and have proven disappointing. This is because many that show promise in the lab, do not then work *in vivo*, have poor bioavailability, cause side effects or act as pro-oxidants (Chaudhary et al., 2023). Compared to small molecules, naturally occurring protein enzymes, which serve as the main antioxidant defence system, are less toxic and up to a million-fold more efficient in eliminating oxidants (Forman and Zhang, 2021).

Our interest in the CLIC proteins as potential cell protective agents has come from both their *in vitro* demonstrated oxidoreductase capabilities (Al Khamici et al., 2015; Alghalayini et al., 2023; Hossain et al., 2025) and their ability to act extracellularly (Hernandez-Fernaud et al., 2017). The ability to act extracellularly was first demonstrated with CLIC3, which was found in the secretome of cancer-associated fibroblast cells (Hernandez-Fernaud et al., 2017). Secreted CLIC3 regulates the binding of transglutaminase-2 (TGM2) to its cofactors, leading to promotion of angiogenesis and cancer progression by promoting TGM2-dependent invasion (Hernandez-Fernaud et al., 2017). The regulation of TGM2 was shown to occur via CLIC3’s oxidoreductase activity, where it enzymatically reduced several of TGM2’s cysteine residues (Hernandez-Fernaud et al., 2017). Recently, we have also highlighted for the first time the role of CLIC proteins as protective antioxidants, when added exogenously at optimal dosage concentrations, to fibroblast and keratinocyte cells in culture (Hossain et al., 2025). We demonstrated that exogenously added recombinant proteins, rCLIC1 or rCLIC4, could protect skin cells from oxidative damage by reducing reactive oxygen species (ROS) production. Furthermore, this ability was dependant on their oxidoreductase activity, as mutation of either of two residues associated with the glutathione S-transferase activity (C24A and K37A) in CLIC1, significantly impaired its ability to protect cells from oxidative stress (Hossain et al., 2025). In addition, we demonstrated that exogenously added rCLIC1 or rCLIC4 provided similar protection against H_2_O_2_-induced oxidative damage compared to either rGrx or rGST-Ω, while the addition of rCLIC3 at varying concentrations, showed no significant impact on skin cell viability, whether in the presence or absence of H_2_O_2_ (Hossain et al., 2025).

Our previous studies have also demonstrated that knockdown of CLIC1 and/or CLIC4, in both human and murine skin cells led to decreased cell viability, increased ROS levels, reduced total cellular oxidoreductase activity, and made the cells more vulnerable to oxidative damage. On the other hand, overexpression of either of the two proteins, afforded cell protection by reducing ROS levels thereby, strongly implicating their involvement in regulating the redox balance in skin cells (Hossain et al., 2025). A recent study by Al Khamici et al. (2023) also showed that CLIC4 supports redox homeostasis and mitochondrial function in 6DT1 breast tumor cells, as knockdown of CLIC4 expression in these cells led to increased ROS and superoxide levels, making the cells more vulnerable to oxidative damage (Al Khamici et al., 2022). Furthermore, there are now a growing number of studies implicating CLIC and related CLIC-like proteins in a variety of redox cellular processes (Hossain et al., 2025; Al Khamici et al., 2022; Do et al., 2016; Liang et al., 2017; Ponnalagu et al., 2022; Ponnalagu et al., 2018; Weston et al., 2021; Zhang et al., 2015). Overall, all these findings support potential roles of CLIC1 and CLIC4 as critical players in regulating ROS levels in cells, emphasizing their contribution to the cellular antioxidant defense system against oxidative stress and in the maintenance of cell health and viability. To further investigate CLIC proteins’ antioxidant capabilities against external environmental stressors, this study aimed to investigate the cellular protective roles of CLIC proteins against UV irradiation-induced damage in human skin cells. UV radiation is a major environmental stressor that accelerates skin aging, DNA damage, and the formation of reactive oxygen species (ROS) (Garg et al., 2020; Wei et al., 2024). In the current study we used a UVC-lamp to expose the cells to UV radiation at a dosage commonly used in the beauty industry. By examining the cellular response to UV-induced oxidative stress, we aimed to uncover how exogenously added CLIC1 and CLIC4 proteins contribute to skin cell resilience, potentially offering new insights into their role as key antioxidants in skin defense mechanisms.

## 2 Materials and methods

The following reagents were purchased from ThermoFisher Scientific (Sydney, Australia): TrypLE™ Express Enzyme (1X) no phenol red; DMEM/F-1; GlutaMAX™ supplement; Opti-MEM™ Reduced Serum Medium; DMEM/F-12, HEPES, no phenol red; Human Fibroblast Medium; Low Serum Growth Supplement (LSGS); EpiLife Medium and Human Keratinocyte Growth Supplement (HKGS). Fetal Bovine Serum (FBS) (French Origin) was purchased from Scientifix. NIH/3T3 murine fibroblast cells were available in-house at UTS. Human Dermal Fibroblasts (HDF) primary cells (C0135C) and Human Epidermal Keratinocytes (HKE) primary cells (C0055C) were purchased from ThermoFisher Scientific (Sydney, Australia). Bacterial streak plates containing CLIC1 in the pIRES2-EGFP Vector (Clonetech) with an amino FLAG (AF-CLIC1) tag and empty construct were generously gifted from the University of New South Wales, Australia. The CLIC4 plasmid (AF-CLIC4), in the same vector, was prepared by Gene Universal as a lyophilized pellet and was transformed into XL1-Blue *E.coli* cells (Agilent Technologies). The following reagents were all purchased from Sigma Aldrich: glutathione reductase (GR) from yeast, reduced glutathione (GSH), bovine plasma thrombin, kanamycin, isopropyl ß-D-1-thiogalactopyranoside (IPTG), tris(2-carboxyethyl) phosphine (TCEP), indanyloxyacetic acid (IAA-94). All other reagents used were of analytical grade.

### 2.1 Cell culture

HDF cells were grown in Human fibroblast media supplemented with LSGS and HKE cells in EpiLife media supplemented with HKGS. NIH/3T3 cells were grown in DMEM/F12 media supplemented with 5% FBS. The cells were incubated at 37 °C 5% CO_2_ and passaged twice a week at a 1:5 dilution to maintain healthy growth. The cells were counted using a T20 cell counter (Bio-Rad) before being seeded in either 96/24/6-well cell culture plates and were grown to ∼90% confluency before being subjected to different biochemical analysis and/or collected to prepare cell lysates for further characterisation. Cells were lysed and stored at −80 °C in RIPA lysis buffer (25 mM Tris HCl, 150 mM NaCl, 0.1%SDS, 1%sodium deoxycholate, 1%NP-40, pH 7.6) and protein concentration was determined using the Pierce ^TM^ BCA Protein Assay Kit (ThermoFisher Scientific) according to the manufacturer’s instructions.

### 2.2 siRNA knockdown of CLIC1 or CLIC4 in NIH/3T3, HDF and HKE cells

Knockdown of CLIC1 or CLIC4 were induced in HDF, HKE and NIH/3T3 cells using transient siRNA-based gene knockdowns using either CLIC1(sc-60400) or CLIC4 (sc-105213) siRNAs (Santa Cruz) along with a Scramble siRNA-A (sc-37007) used as a control as previously described (Hossain et al., 2025). For ease of understanding, CLIC1 or CLIC4 knockdown cells are annotated as CLIC1-KD and CLIC4-KD respectively, CLIC1 and CLIC4 Double Knockdown cells as DKD and the scramble siRNA control as Scmb C. Briefly, transfection was carried out using Lipofectamine siRNAMAX (ThermoFisher Scientific) according to the manufacturer’s instructions with the exception that a 20 pmol total concentration of siRNA was used for HDF and HKE cells (as concentration above that showed toxicity) to produce either CLIC1-KD or CLIC4-KD cells and 10 pmol of each CLIC1 and CLIC4 siRNA used for the DKD cells. Successful knockdown was then assessed via Western blot analysis of the cell lysates using rabbit anti-CLIC1 (mAb-53424, Cell Signaling) and mouse monoclonal anti-CLIC4 (sc-135739, Santa Cruz) antibodies with β-Actin (Invitrogen) used as the loading control (data previously shown in Hossain et al., 2025). The day prior to transfection, cells were seeded at a cell density of 2.5 × 10^4^ cells per well in a six well plate and incubated over-night at 37 C 5% CO_2_ in their respective media. siRNA Transfected cells were allowed to grow for a period of 3 days, thrice washed with PBS before they were subjected to different biochemical analysis and/or collected to prepare cell lysates for further characterisation.

### 2.3 Transfection of NIH/3T3 cells for CLIC1 or CLIC4 overexpression

NIH/3T3 cells were transfected with either CLIC1 (AF-CLIC1), CLIC4 (AF-CLIC4) or empty pIRES2-EGFP vector as previously described (Hossain et al., 2025). Briefly, harvested NIH/3T3 cells were transferred to Gene Pulser 0.4 cm Cuvettes (Bio-Rad) to which 5 µg of DNA plasmid (either AF-CLIC1, AF-CLIC4 or vector control pIRES2-EGFP) were added to each respective cuvette with the addition of 10 µL 1M HEPES Buffer, pH 7.2 (Gibco). The cells were electroporated using the GenePulser MXCell Unit (Bio-Rad), harvested by centrifugation and resuspended in sorting buffer (PBS containing 5% FBS and 5 mM EDTA, pH 7.2). The cells were then analysed and sorted on the BD Influx (BD Biosciences) into 96 well collection plates for the growth of stable EGFP + cells overexpressing either CLIC1 or CLIC4. For experimental purposes, stable overexpressing NIH/3T3 cells transfected with CLIC1 (AF-CLIC1), CLIC4 (AF-CLIC4) or empty pIRES2-EGFP vector were grown to ∼90% confluency before being subjected to different biochemical analysis and/or collected to prepare cell lysates for further characterisation. Overexpression of the proteins were confirmed using Western blot analysis using the respective antibodies as previously shown in (Hossain et al., 2025).

### 2.4 CLIC1 or CLIC4 recombinant protein expression and purification

Glycerol stocks of ClearColi BL21 (DE3) cells transformed with the His-tagged PET28a (+) expression vector (Novagen) containing the coding sequence for either human CLIC1 (NP_001279) or CLIC4 (NP_039234) were used to express either rCLIC1 or rCLIC4 recombinant proteins by methods as previously described (Alghalayini et al., 2023). Briefly, cells expressing the recombinant CLIC proteins (rCLIC1 or rCLIC4) were grown in 2xYT medium containing 30 μg/mL kanamycin (Sigma Aldrich) and were then induced with 1 mM IPTG (Sigma Aldrich) at 20 C with overnight shaking at ∼180 rpm. Cells were then harvested and the His-tagged rCLIC proteins were purified via affinity chromatography using a Ni^2+^-NTA (Qiagen) column. The His-tag was removed by in-column thrombin enzymatic cleavage using an overnight incubation of bovine plasma thrombin (Sigma Aldrich) (30 NIH units per 1 L of bacterial culture) at 4 C. The cleaved rCLIC proteins were then collected in PBS buffer (10 mM phosphate buffer, 2.7mM KCl, 140 mM NaCl, pH 7.4, and 0.5 mM TCEP) and further purified through size exclusion chromatography (SEC) (AKTA Pure/Amersham Pharmacia Biotech) using a HiPrep™ 16/60 Sephacryl® S-100HR column (Sigma Aldrich) and equilibrated in column sizing buffer (100mM KCl, 20 mM HEPES, pH 7.2). rCLIC proteins purity was verified via Western blot analysis using their respective anti-CLIC antibodies (Santa Cruz) and anti-His antibody. Protein concentrations were measured using the Pierce™ BCA Protein Assay Kit (ThermoFisher Scientific) according to the manufacturer’s instructions and functional activity was assessed using the HEDS assay (details below). The purified samples were then aliquoted and stored at −80 °C for future experiments.

### 2.5 Effects of rCLICs on the viability of human skin cells challenged with UV irradiation

To determine any possible cellular protective and antioxidant roles of CLICs, cells were treated with recombinant rCLIC proteins for 1 h prior to being exposed to UV irradiation. Cells were first treated with rCLICs at their respective optimal dosage concentration in media without supplement (no FBS) for 1 h and then washed thrice to remove any exogenously added recombinant protein from the well. The cells were then exposed to 30 min of UV irradiation at a calculated dosage amount. The Half-maximal inhibitory concentration (IC_50_) of UV exposure was determined prior to this experiment on both HDF and HEK cells (data shown in Supplementary Figure S1) resulting in approximately 25% cell damage caused after 30 min UV exposure. In order to expose the cells to UVC at a calculated dosage amount, a Super SunUV lamp (SunUV) at a dose of 2.175 × 10^13^ J*m-2*sec-1 was used. To calculate the dosage/fluence (J*m-2*sec-1), the following Equation 1 was used and modified accordingly as shown in Equations 2, 3:

$$I=P/A=P/\left(4*\pi *r2\right)$$ (1)

where A is the area of the sphere of radius r (i.e., A = 4π*r2), P is the output of the lamp in Watts (W) and I is the intensity across the entirety of the surface r. Because the samples were seeded in 96 well plates, to determine the area per well, the ratio: (a/A) is taken into consideration where a is the surface area of the well. As a result, the intensity I should be represented as Is which is represented as:

$$\text{Is}=I*\left(a/A\right)$$ (2)

In order to determine the dosage or fluence (J*m-2*sec-1), Is must be divided by the energy Eph of the photons (where Eph = c*h/λ, with c being the speed of light (3 × 10^8^ m/s), h is the Planck constant (6.63 × 10^−34^ J) and λ is the wavelength of the UV lamp (2.54 × 10^7^ m)). Hence the final equation is:

$$\text{Fluence}=\text{Is}/\text{Eph }\left(J*m-2*\sec-1\right)$$ (3)

Cells not treated with rCLICs or subjected to UV exposure; cells not treated with rCLICs but subjected to UV exposure and cells treated with rCLICs but not subjected to UV exposure were used as experimental controls. Following different treatments, cell viability was measured using the WST-1 reagent as mentioned below. To indirectly demonstrate that the change in cellular activity is likely associated with rCLIC protein activity, the drug IAA-94 at a concentration of 1 μM was pre-incubated with the recombinant proteins for an hour on ice before being added to the cells. For each experiment, data was collected from three different passages with each passage ran in triplicate for all the cell types.

### 2.6 Cell viability assay

Cell viability was measured using a colorimetric assay for 96/6-well plates with 2-(4-iodophenyl)-3-(4-nitrophenyl)-5-(2,4-disulfophenyl)-2H-tetrazoliummonosodium salt (WST-1) reagent according to the manufacturer’s instructions as previously described (Hossain et al., 2025). Briefly, all the different cells, including siRNA CLIC knockdown cells or stable overexpressing CLIC cells or rCLICs treated cells and the controls, following different treatments were washed with PBS thrice and incubated with 10% WST-1 reagent for a period of 4 h. Cell viability was measured using a TECAN-Infinite M1000 microplate reader at 440 nm with reference wavelength at 600-nm. Data was analysed using GraphPad Prism 10 and represented as percentage cell viability in comparison to that of the non-treated cells (cells not subjected to any sort of treatment). Data is also represented as percentage cellular protection where the difference in cell viability is compared between the rCLIC treated and Control cells exposed to UV irradiation. For each experiment, data was collected from three different passages with each passage run in triplicate for each cell types.

### 2.7 Measuring reactive oxygen species (ROS) levels

To detect the levels of Reactive Oxygen Species (ROS), a fluorescent cellular reactive oxygen species detection assay kit (Red Fluorescence) (Abcam) was used according to the manufacturer’s instructions as previously described (Hossain et al., 2025). Cells were seeded at a density of 2 × 10^4^ cells per well in 96 flat black well plates (Corning) and incubated at 37 C, 5%CO_2_ overnight. The following day, the different types of cells were subjected to different treatments after which the fluorescent stain was applied and immediately read using the TECAN-Infinite M1000 microplate reader at Ex/m = 520/605 nm. For each experiment, data was collected from three different passages with each passage ran in triplicate.

### 2.8 Characterisation of antioxidant activity of exogenously added rCLICs in comparison to well-known antioxidants

HDF cells were plated and treated as described above with the exception that the cells were treated with either rCLICs or with equimolar concentration of enzymatic antioxidants like Glutaredoxin (Grx), GST-Omega (GST-Ω) such that the total protein concentration is 0.15μM and 4 mM of N-acetylcysteine (NAC) before being subjected to either no treatment (no UV) or 30 min UV irradiation (+UV). For each experiment, data was collected from three different passages with each passage ran in triplicate.

### 2.9 HEDS assays using whole cell lysates of HDF and HKE cells

In order to determine changes in the oxidoreductase activity, whole cell lysates collected from the different cells (including rCLIC treated and siRNA CLIC knockdown cells) following different treatments and their respective controls were subjected to HEDS assay. All HEDS enzyme assays were performed in a flat 96-well plate containing 10 µg final protein concentration added to a potassium phosphate buffer (5 mM/pH 7) that contained 1 mM EDTA, 250 µM NADPH, 1 mM HEDS, and 0.5 μg/mL GR. The mixture was incubated for 5 min at 37 C, and the enzymatic reaction was initiated by the addition of 1 mM GSH to make up a final volume of 200 μL in each well. The consumption of NADPH was measured at A340 nm using the TECAN-Infinite M1000 microplate reader. Statistical analysis was performed using Two-way Anova with Turkey’s comparison and are presented as the Mean ± SEM. For each HEDS assay, cell lysates were collected from three different passages with each passage run in triplicate for each of the different types of cells. To indirectly demonstrate that the change in oxidoreductase activity is likely associated with CLIC protein activity, 10 μM of IAA-94 was pre-incubated with the whole cell lysates for an hour on ice before being added to the HEDS assay mix. Buffer with or without 10 μM IAA-94 was used as control to ensure that the drug itself does not interfere with the assay.

### 2.10 Detection of reactive oxygen species markers using western blot

In order to detect changes in the expression levels of different cellular oxidative stress markers, the following antibodies were used: Catalase (sc-271803), Thioredoxin (Trx, sc-271281), Nitrotyrosine (sc-32757), Superoxide Dismutase type 1 (SOD1, sc-101523) (all antibodies were from Santa Cruz and used in a 1:1000 dilution in PBS-T), with β-Actin (Invitrogen) used as the loading control at a 1:2500 dilution. 10ug whole cell lysates of CLIC1 or CLIC4 treated or single or double siRNA knockdown cells and their respective controls were loaded into a Mini-PROTEAN TGX Gel (Bio-Rad) and ran at 170V for 40 min in 1x TRIS/Glycine/SDS running buffer. The gels were then transferred to PVDF membrane using the TurboBlot (Bio-rad). The membranes were removed from the cassette and blocked for 1 h in 5% bovine serum albumin in PBS-T (PBS +0.5% Tween) blocking solution. The membranes were incubated with the different antibodies overnight at 4 C with rolling. The following day, the membranes were washed with PBS-T and incubated in a goat-anti-mouse secondary antibody (Invitrogen) for 2 h and then imaged using the ChemiDoc and Clarity ECL. Densitometry analysis was conducted in Fiji/ImageJ and further processed in GraphPad Prism 8 (GraphPad Software, La Jolla California USA).

### 2.11 Effect of rCLICs treatment on either CLIC1 or CLIC4 siRNA knockdown HDF cells

To better understand the effects of rCLICs treatment, knockdown of CLIC1 or CLIC4 were induced in HDF cells using transient siRNA-based gene knockdowns as described above. siRNA CLIC1-KD/CLIC4-KD cells were incubated with either rCLIC1 or rCLIC4 in HDF media (no supplement) for 1 h followed by either no treatment or 30 min UV irradiation. Cell viability was determined using the WST-1 reagent (Sigma Aldrich) according to the manufacture’s instruction.

## 3 Results and discussion

### 3.1 CLIC1 or CLIC4 knockdown or over-expression impacts on the viability and ROS levels of murine NIH/3T3 cells and their susceptibility to damage following UV exposure

**FIGURE 1:**
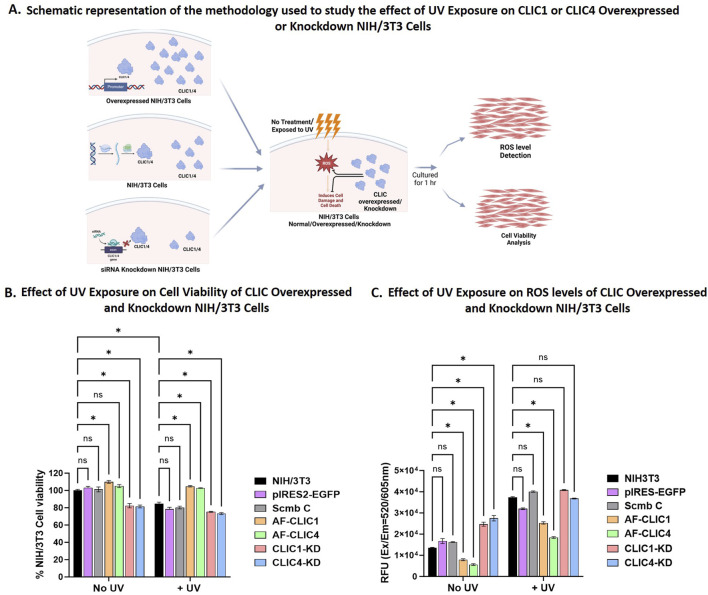
Cell viability and ROS levels for NIH/3T3 cells overexpressing or Knockdown for CLIC1 or CLIC4. **(A)** Schematic representation of the methodology used to study the effect of UV exposure on CLIC1 or CLIC4 Overexpressed and Knockdown NIH/3T3 cells. Created in BioRender. Hossain, K. (2025) https://BioRender.com/y3sy5mo. **(B)** Examining Changes in Cell Viability after UV Exposure for 30 min. **(C)** Examining changes in cellular ROS Levels after UV Exposure for 30 min. In each figure, the results are shown for overexpressing cells: AF-CLIC1 (orange) and AF-CLIC4 (green); siRNA knockdown cells: CLIC1-KD (red) and CLIC4-KD (blue) and the three control cell lines: NIH/3T3 (black), empty vector pIRES2-EGFP (purple) and Scmb C (silver). Data expressed as Mean ± SEM. Two-way ANOVA with Tukey’s multiple comparisons test was done. *P < 0.05, Data was collected from three different passages with each passage run in triplicate.

Previously we have shown that overexpressing CLIC1 or CLIC4 in NIH/3T3 imparted cellular protection via their oxidoreductase activity against H_2_O_2_-induced oxidative damage, whereas their knockdowns were more suseptible to oxidative damage and was associated with incraesed ROS levels (Hossain et al., 2025). To specifically examine the cellular protective roles of CLIC1 and CLIC4 against UV induced cellular damage, we generated individual stable CLIC1 (AF-CLIC1) or CLIC4 (AF-CLIC4) over-expressing cell lines, as well as, transient CLIC1 (CLIC1-KD) or CLIC4 (CLIC4-KD) siRNA knockdown using NIH/3T3 cells, and compared their activity to their respective controls (pIRES2-EGFP, Scmb C and NIH/3T3 cells), a schematic representation of the method is shown in Figure 1A and the results shown in Figures 1B,C. A number of attempts to overexpress either protein in primary human skin cells (HDF and HKE) were not successful, therefore, only data for murine NIH/3T3 cells were obtained and shown below.

In order to validate the protective roles of CLIC proteins, cells were exposed to UV irradiation at a calculated dosage for a period of 30 min. This empirically pre-determined dosage ensured UV-induced cell damage that resulted in a reduction in total cell viability by no greater than 25% (data shown in Supplementary Figure S1). Changes in cell viability between the overexpressing or knockdown cells and the controls were measured using WST-1 reagent and data shown in Figure 1B. UV irradiation was detrimental to the CLIC1-KD and CLIC4-KD cells, resulting in approximately 25% ± 0.89% and 27% ± 1.75% cell damage respectively, in comparison to only 10% ± 3.33% cell damage shown by the three different Control cell lines, with no significant difference seen between the controls: NIH/3T3 cells, Scmb C and pIRES2-EGFP. No significant difference in cell viability following UV exposure was seen between the CLIC1-KD and CLIC4-KD cells, suggesting that knockdown of either CLIC1 or CLIC4 results in NIH/3T3 cells being equally susceptible to UV damage. Both CLIC1-KD and CLIC4-KD showed significant reduction in cell viability in the absence of any UV exposure (No UV), with no visible difference between the Control cell lines. This thereby strongly suggests that the cellular damage seen in the absence or presence of UV irradiation in the knockdown cells, was likely induced by the reduced CLIC1 or CLIC4 expression.

On the other hand, NIH/3T3 cells overexpressing either CLIC1 (AF-CLIC1) or CLIC4 (AF-CLIC4) showed significant increase in cell viability in comparison to the Control cell lines upon exposure to UV irradiation. Both CLIC overexpressing cells were more resilient to UV damage, resulting in almost 100% cell viability, with no significant difference seen between the treated (+UV) and the non-treated (No UV) CLIC1 or CLIC4 overexpressing cells. This clearly shows that murine fibroblast cells overexpressing either CLIC1 or CLIC4 proteins are either less susceptible to UV damage or are protected against UV damage, in comparison to their counterpart knockdown and control cell lines. This cellular protective role and the antioxidant ability of CLIC1 and CLIC4 against UV damage was also evident from Figure 1C.

In the presence of UV irradiation, cells overexpressing either CLIC1 or CLIC4 resulted in approximately 32% and 50% decrease in ROS levels respectively in comparison to the Control cell lines. Of note, in the absence of UV irradiation, AF-CLIC1 and AF-CLIC4 cells also displayed significantly lower levels of native ROS. On the other hand, upon UV irradiation, CLIC1-KD and CLIC4-KD cells showed a significant 3-fold increase in their ROS levels, which was similar to the Control cell lines. There were no significant differences seen between the CLIC1 or CLIC4 knockdown cells against the Control cell lines, however, CLIC1-KD cells had significantly higher ROS levels compared to CLIC4-KD cells upon exposure to UV irradiation. In the absence of UV irradiation, however, significant differences were found, with the knockdown cells having approximately 60% ± 5% higher basal ROS levels compared to the controls, with no significant difference seen between the CLIC knockdown cells, unlike that observed in the presence of UV irradiation.

### 3.2 Exogenous addition of rCLIC1 or rCLIC4 or knockdown of either protein, impacts on the cell viability and ROS levels of primary HDF and HKE cells and their susceptibility to damage following UV exposure

**FIGURE 2:**
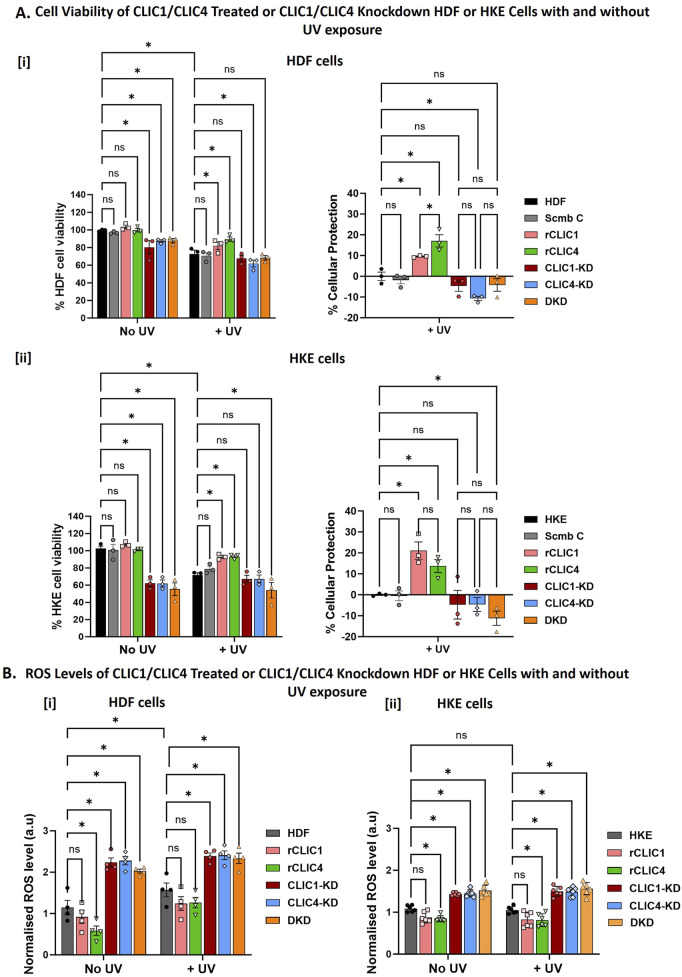
Percentage cell viability, Percentage cellular protection and ROS levels following rCLICs treatment or siRNA CLIC knockdown of HDF and HKE Cells With/Without UV exposure **(A)**. Examining Changes in Cell Viability and the corresponding Percentage Cellular protection following recombinant rCLIC1 or rCLIC4 treatment or siRNA knockdown of either CLIC1/4 and their Double Knockdown prior to UV Exposure for 30 min in [i] HDF and [ii] HKE cells. **(B)** Examining changes in cellular ROS Levels after UV Exposure for 30 min in [i] HDF and [ii] HKE cells. In each figure, the results are shown for recombinant CLIC treated HDF or HKE cells: rCLIC1 (pink) and rCLIC4 (green); siRNA knockdown cells: CLIC1-KD (red), CLIC4-KD (blue) and CLIC1 and CLIC4 Double Knockdown (DKD, orange). The control which are non-treated HDF/HKE cells are shown in grey. And in **(A)** only, the Scramble Control (Scmb C) treated with equimolar scramble siRNA is shown in silver. Data expressed as Mean ± SEM. Two-way ANOVA with Tukey’s multiple comparisons test was done. *P < 0.05, Data was collected from three different passages with each passage run in triplicate.

Previously, we have demonstrated for the first time, the protective antioxidant properties of purified recombinant CLIC proteins when added to cells in culture (Hossain et al., 2025). Dosage optimisation of rCLICs for HDF and HKE cells resulted in optimal concentrations of 0.15 µM for rCLIC1 or 0.2 µM for rCLIC4, when added exogenously. At these concentrations, neither recombinant protein impacted negatively on cell viability nor induced cell proliferation, while showing cellular protection against H_2_O_2_-induced oxidative damage by reducing ROS levels and increasing cell viability in both HDF and HKE cells (Hossain et al., 2025). On the other hand, knockdown of either protein was found to be detrimental to HDF and HKE cells, and subsequent treatment of these human skin cells with H_2_O_2_, resulted in increased susceptibility to H_2_O_2_-induced oxidative stress and greater levels of damage (Hossain et al., 2025). In the current study, we used that same optimal concentration of rCLIC1 (0.15 µM) and rCLIC4 (0.2 µM) to study their cellular protective capabilities against UV-induce damage and comparable results were also observed in the absence and presence of UV irradiation as seen in Figure 2. Knockdown of either CLIC1 (CLIC1-KD), CLIC4 (CLIC4-KD) or Double-Knockdown (DKD), all resulted in cellular damage that significantly reduced the cell viability in both HDF and HKE cells compared to control cells, with no significant differences seen between any of the three knockdowns. In HDF cells, CLIC1-KD, CLIC4-KD or DKD caused a reduction in cell viability by approximately 20% ± 7.5%, 23% ± 6% and 16% ± 4.18% respectively. While, the human keratinocyte cells were clearly more sensitive to CLIC knockdowns, resulting in a reduction of 41% ± 10.4%, 38% ± 9.4% and 45% ± 12.8% for CLIC1-KD, CLIC4-KD or DKD respectively. On the other hand, exogenous addition of CLIC recombinant proteins, (at optimal dosage concentrations of 0.15 µM for rCLIC1 or 0.2 µM for rCLIC4 to HDF or HKE cells in culture), showed no significant difference in comparison to the non-treated Control cells, thereby suggesting that, unlike downregulation, recombinant CLIC1 or CLIC4 treatment has no obvious deleterious effects on these cells, nor did it cause cell proliferation when used at these concentrations. These results are in agreement with similar studies previously published (Hossain et al., 2025).

In order to then assess the cellular antioxidant and protective roles of CLICs against UV-induced damage, CLIC1 or CLIC4 single or double knockdown cells and rCLIC1 or rCLIC4 treated cells were exposed to UV irradiation for a period of 30 min. Cell damage was confirmed by a reduction in <25% cell viability in control cells following UV exposure (Figure 2A left). It is clearly evident from Figure 2Ai that HDF cells treated with either rCLIC1 or rCLIC4 for 1 h prior to UV exposure resulted in a % cellular protection of approximately 10% ± 0.5% and 17.1% ± 5.1% respectively in comparison to the UV treated control cells (HDF) with rCLIC4 providing significantly greater protection compared to rCLIC1 treatment. Similar protection was also observed following exogenous addition of rCLIC1 or rCLIC4 to HKE cells (Figure 2Aii), also resulting in a % cellular protection of approximately 21% ± 7.2% and 14% ± 5.3% respectively, in comparison to the UV treated control cells (HKE) with no significant difference seen between the two recombinant protein treatments.

Total cellular ROS was measured to assess if the protein protection seen following rCLIC treatment, was linked to changes in the cellular ROS levels, results shown in Figure 2B. HDF (Figure 2Bi) and HKE (Figure 2Bii) cells, not subjected to UV irradiation (No UV) but treated with either rCLIC1 or rCLIC4, both showed a small reduction in basal ROS levels, but only those treated with rCLIC4 showed a significant decrease. Similarly, upon exposure to UV irradiation, pre-treating the skin cells with either of the rCLICs also showed a small, yet non-significant difference in the ROS levels, except for rCLIC4 treatment of HKE cells that showed a significant 0.3-fold decrease in ROS levels in comparison to the Control (non-treated) cells. This was somewhat unexpected, given that previously we had shown both rCLIC1 and rCLIC4 pre-treatment of HDF and HKE cells resulted in significant reduction in ROS levels when the cells were subsequently exposed to H_2_O_2_ (Hossain et al., 2025). Even though both UV (Schuch et al., 2017), and H_2_O_2_ (Chidawanyika and Supattapone, 2021) can result in oxidation of cellular macromolecules, formation of free radicals and DNA damage, the extent of the damage caused by either, as well as, their respective modes of action may differ. This point may partly explain the different outcomes seen in the levels of ROS when comparing UV exposure versus chemically induced oxidative stress. Another possible explanation might be that CLIC1 and CLIC4 act independently of each other, operating via distinct signaling pathways and/or distinct substrate targets, following either H_2_O_2_ or UV exposure, that may or may not involve directly regulating cellular ROS levels. Such details remain to be clarified and warrant further investigation and were beyond the scope of the current study. Despite this, it is evident from these results that overexpressing CLIC proteins in murine fibroblast cells (Figure 1B) and exogenously treating human fibroblast (Figure 2Ai) and keratinocyte cells (Figure 2Aii) with either rCLIC1 or rCLIC4 protein, not only imparts cellular protection against H_2_O_2_-induced oxidative stress, as previously shown (Hossain et al., 2025), but also provides protection against UV-induced cellular damage.

Conversely, UV irradiation of CLIC1-KD, CLIC4-KD and DKD (in either HDF or HKE) cells resulted in a reduction in cell viability when compared to non-UV treated control cells. This was also evident from the negative % cellular protection values seen for the UV-treated knockdown cells in comparison to the UV-treated Control cells (HDF/HKE) and scramble (Scmb C) Control cells. Despite this observed trend in reduction in cell viability, there were no significant differences seen between the knockdown and the control cells; with the exception that CLIC4-KD in HDF and DKD in HKE cells showing significant reductions. Comparison of the cell viability and the percentage cellular protection found that there were no significant differences between the CLIC1-KD, CLIC4-KD and DKD for both HDF and HKE cells following UV exposure. This was interesting because unlike with the H_2_O_2_ treatment (Hossain et al., 2025), the CLIC knockdown human skin cells were no more susceptible to UV-damage nor did it induce greater cell damage, compared to the UV-treated control cells. This was also evident from the ROS levels as seen in Figure 2B. In the absence of UV irradiation, CLIC1-KD, CLIC4-KD and DKD cells all showed significant increases in their basal ROS levels in comparison to the controls, with no significant difference seen between the three different knockdown cells. Knockdown of either CLIC1, CLIC4 or Double-knockdown in HDF cells resulted in approximately a 2-fold increase, while HKE showed an estimated 1.3-fold increase, in basal ROS levels. However, no significant difference was observed in the ROS values for the different treatments in both HDF and HKE cells following UV exposure.

### 3.3 Assessment of the antioxidant capability of r CLICs compared to other known antioxidant protective agents

Previous studies have shown exogenous addition of well-known enzymatic antioxidants to cells, like superoxide dismutase (SOD), catalase, glutathione peroxidase (Gpx) and Grx, protected the cells from oxidative damage (Aksoylar and Patsoukis, 2023; Bernier et al., 1989; Bonetta, 2018; Chen et al., 2013; Del Prete et al., 2019; Mao et al., 1993; Tominaga et al., 2012). We have also confirmed using fluorescent staining that exogenous addition of rCLICs resulted in the intracellular uptake of the recombinant proteins by HDF and HKE cells which then imparted protection against H_2_O_2_-induced oxidative damage and showed antioxidant capabilities (Hossain et al., 2025). Thus far, results from this study also strongly support the cellular protective roles of CLIC1 and CLIC4 against UV-induced damage. Therefore, to further characterise their roles as antioxidants, their activity was compared to that of well-known enzymatic antioxidants.

**FIGURE 3:**
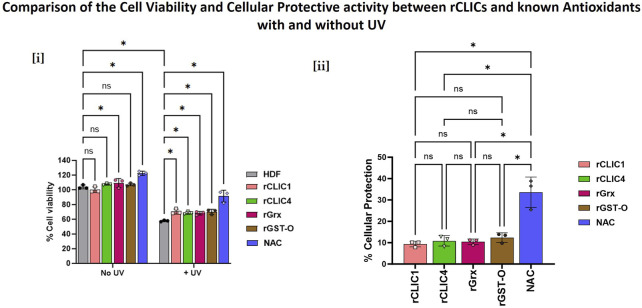
Comparison of the percentage cellular protection shown by different rCLIC treatments in HDF cells against the following known antioxidants: Grx, GST-O, and NAC. Cells were treated with equimolar enzymatic antioxidants (0.15 μM) or NAC (4 mM) for 1 h prior to UV exposure for 30 min. Cell viability was measured using WST-1 reagent. [i] Data represented as the percentage cell viability in comparison to the Control shown by the different antioxidants with or without UV exposure. [ii] Data was further analysed to determine the percentage cellular protection shown by the different antioxidants against UV-induced damage. Data expressed as Mean ± SEM. Two-way or One-way ANOVA with Tukey’s multiple comparisons test was done. *P < 0.05, Data was collected from three different passages with each passage run in triplicate.

HDF cells were treated with equimolar amounts of rCLIC1, rCLIC4 and known endogenous protein antioxidants including rGrx (abcam, ab86987) or rGST-Ω (Merck, GS75) at a final protein concentration of 0.15 μM. HDF cells were also treated with the chemical antioxidant N-acetyl cysteine (NAC) at a concentration of 4 mM as this was shown to be the optimal antioxidant concentration for NAC based on previously published studies (Ezeriņa et al., 2018; Liu et al., 2019; Montero et al., 2023). The cells were treated with the different proteins or NAC for 1 h before being subjected to UV irradiation for 30 min after which cell viability was measured using WST-1 reagent. The difference in cell viability between the Control cells (not treated with any antioxidants) and the treated cells after being exposed to UV irradation and the percentage cellular protection imparted by the different treatments are shown in Figure 3. As seen in Figure 3i, cells treated with rCLICs or other known antioxidants prior to UV exposure, all resulted in significant increases in cell viability following UV exposure in comparison to the Control cells that received no prior antioxidant treatment. Both rCLIC1 and rCLIC4 protected HDF cells against UV-induced damage, resulting in a percentage cellular protection of approximately 9.38% ± 1.3% and 11% ± 2.4% respectively as shown in Figure 3ii. These results are comparable to those of the other well-known enzymatic antioxidants (Hossain et al., 2025). Grx and GST-Ω showed similar cellular protection of 10.4% ± 1.3% and 12.4% ± 2.26% respectively, while the small molecule antioxidant NAC, showed the greatest amount of protection of around 33.6% ± 7.14%. These results demonstrate that equimolar amounts of rCLIC1 or rCLIC4 is capable of providing cellular protection at levels comparable to equimolar amounts of either recombinant GST-Ω or Grx proteins; thus, further validating the role of rCLICs in regulating the redox environment of skin cells. Whether the rCLICs provide their cell protection via a similar mechanism to the Grx and GST-Ω proteins, warrants further investigation.

### 3.4 Effect of CLIC1 or CLIC4 treatment or knockdown on the total oxidoreductase activity of HDF and HKE cells following UV exposure

**FIGURE 4:**
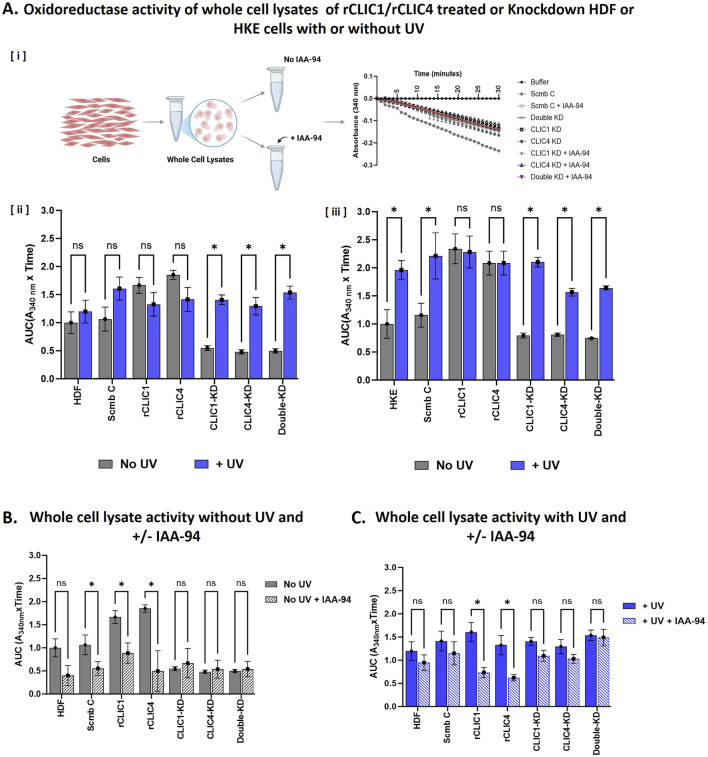
**(A)** Oxidoreductase activity and the corresponding Area Under the Curve (AUC) of whole cell lysates following rCLIC treatment or siRNA knockdown. The cells were either pre-treated with rCLIC1 or rCLIC4 for 1 h or pre-treated with siRNAs to generate single or double knockdowns: CLIC1-KD, CLIC4-KD and DKD. Both the rCLIC treated and knockdown cells were exposure to UV irradiation for 30 min and then the cell lysates were collected and either not pre-incubated or pre-incubated with the drug IAA-94 for 1 h before being subjected to HEDS assay. Shecmatic reprentation of the methodology is shown in [i] and HDF [ii] or HKE [iii] cells not exposed to UV (No UV) are shown in silver and cells that were exposed (+UV) are shown in blue. Cell lysates collected from HDF cells following no treatment with UV **(B)** or exposed to UV **(C)** and then pre-incubated without or with IAA-94 are represented as solid or stripped bars respectively. Two-way ANOVA with Tukey’s multiple comparisons test was done. *P < 0.05, ns = not significant. Data represented as Mean ± SEM. For each experiment, data was collected from three different passages with each passage run in triplicate. Schematic representation of the methodology was created in BioRender. Hossain, K. (2025) https://BioRender.com/y3sy5mo.

The HEDS enzyme assay has been used extensively in the characterisation of the glutaredoxin family oxidoreductase activity (Al Khamici et al., 2015; Hernandez-Fernaud et al., 2017; Holmgren and Björnstedt, 1995). Using both purified recombinant CLICs (Al Khamici et al., 2015; Alghalayini et al., 2023; Turkewitz et al., 2021) and whole cell lysates (Hernandez-Fernaud et al., 2017; Hossain et al., 2025), we have previously demonstrated the oxidoreductase activity of the CLIC family members in the HEDS enzyme assay. In order to measure changes in the total cellular oxidoreductase activity, whole cell lysates from siRNA knockdown and rCLIC pre-treated HDF or HKE cells, with UV (+UV) or without UV exposure (No UV), were collected and subjected to the HEDS assay analysis. Samples exposed to the CLIC blocker drug IAA94 for 1 h prior to being assayed via the HEDS assay were also included. Figure 4 shows the oxidoreductase activity of the whole cell lysates from HDF and HKE cells and the corresponding area under the curve calculation following no UV treatment or UV exposure in the absence or presence of the drug IAA-94.

Previously, we have shown that lysates collected from CLIC1-KD, CLIC4-KD and Double-KD cells all showed significant decrease in their oxidoreductase activity in comparison to the control cells (expressing both CLIC1 and CLIC4) for both human and murine fibroblast cells and human keratinocyte cells in culture, strongly suggesting CLIC1 and CLIC4 contributes to the total oxidoreductase activity of the cells (Hossain et al., 2025). Similar results were seen in Figure 4A, where in the absence of UV irradiation, all three knockdown cell types showed a significant 2-fold decrease in their oxidoreductase activity for both HDF and HKE cells. While, the rCLIC1 and rCLIC4 treated cells showed significant increase in their oxidoreductase activity, resulting in approximately 1.5-fold increase in their respective AUC values in HDF cells (Figure 4Ai) and approximately 2-fold increase in HKE cells (Figure 4Aii). Samples that were pre-incubated with the drug IAA-94 (Figure 4B), demonstrate an expected decrease in their enzymatic activity, including the control cells that also constitutively express both native CLIC1 and CLIC4, as well as the rCLIC treated HDF cells. On the other hand, there was no significant difference seen in the total oxidoreductase activity for the different knockdown cells when treated with IAA-94. Similar results were also seen for HKE cells (see Supplementary Figure S2). This strongly suggests that the total cell oxidoreductase activity is associated with CLIC expression where downregulation of CLIC1 and/or CLIC4 reduces their activity, while exogenous addition of rCLICs results in their cellular uptake that enhances the activity, which in-turn is inhibited by the drug IAA-94.

On the other hand, comparison of the oxidoreductase activity between the non-treated (No UV) and the UV irradiated (+UV) cells, showed significant increase in the oxidoreductase activity of the knockdown cells, with CLIC1-KD and CLIC4-D showing approximately a 2.5-fold increase while DKD resulted in a 3-fold increase in their activity in HDF cells following UV exposure (Figure 4Ai). Similarly, in HKE cells, CLIC1-KD, CLIC4-KD and DKD also showed a significant increase, between two and 2.5-fold, in activity in comparison to cell lysates not exposed to UV (Figure 4Aii). Control cells also showed increased oxidoreductase activity in HDF and HKE cells following UV irradiation. Interestingly, in the presence of IAA-94 and UV (Figure 4C), there were no significant differences seen in the oxidoreductase activity in any of the knockdown cells or the controls in HDF cells whereas, following UV exposure, HKE control whole cell lysates pre-incubated with IAA-94 showed significant decrease in oxidoreductase activity and CLIC1-KD cells also showed significant decrease in activity when pre-incubated with IAA-94 drug (Supplementary Figure S2B). It is important to note that the cell lysates contain a plethora of other oxidoreductase enzymes (most notably Grxs and Trxs) that can be detected by the HEDS assay and are likely contributing to the total oxidoreductase activity measured in the whole cell lysates. Furthermore, IAA-94 may also be inhibiting other non-CLIC oxidoreductase proteins, therefore these results need to be interpreted accordingly and warrant further investigation. As such, these findings suggest that upon UV irradiation, cells, in particular the CLIC knockdown cells, are likely expressing other cellular oxidoreductase protective proteins to overcome the oxidative stress and/or damage induced by UV and these proteins are likely contributing to the overall oxidoreductase activity of the cells and are thus not inhibited by the CLIC blocker, IAA-94. Whereas, both HDF and HKE cells treated with rCLICs showed no significant difference in their oxidoreductase activity following UV exposure and their activity was significantly reduced by the drug IAA-94, similar to that seen in the absence of UV. These results clearly indicate that the oxidoreductase activity of CLIC1 and CLIC4 proteins play a crucial role in protecting cells against UV-induced damage and down-regulating the CLICs most likely stimulates the expression of other protective proteins, to compensate and assist with combating UV-induced damage. In order to further validate these findings and to elucidate specific changes in the relative expression levels of other oxidative stress related proteins, Western blot analysis of whole cell lysates were undertaken, as outlined below.

### 3.5 Determining oxidative stress related protein expression changes following exogenous addition of rCLIC1 or rCLIC4 to HDF cells in culture

**FIGURE 5:**
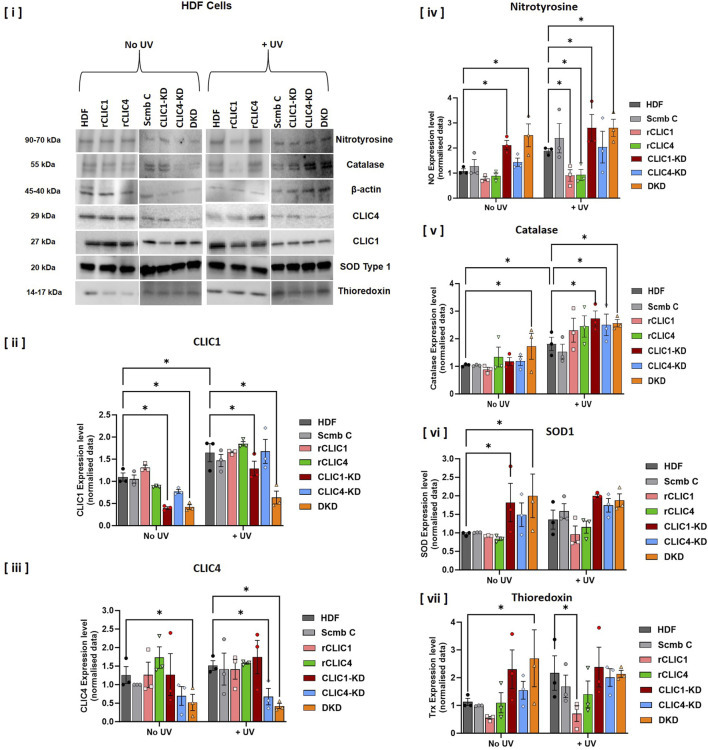
Western blot and densitometry analysis of CLIC siRNA knockdowns or rCLICs treated HDF whole cell lysates either non-treated (No UV) or exposed to 30 min of UV irradiation (+UV). [i] Western blot sections showing different antibody staining from one representative gel. Densitometry analysis for each of the different antibodies as shown in the subsequent figures [ii] CLIC1; [iii] CLIC4; [iv] Nitrotyrosine; [v] Catalse; [vi] SOD1 and [vii] Thioredoxin. In each figure, the results are shown for recombinant CLIC treated HDF cells: rCLIC1 (pink) and rCLIC4 (green); siRNA knockdown cells: CLIC1-KD (red), CLIC4-KD (blue) and CLIC1 and CLIC4 Double Knockdown (DKD, orange). The controls either non-treated HDF cells are shown in grey and the Scramble Control (Scmb C), treated with equimolar scramble siRNA, is shown in silver. 10 μg of protein was loaded for each sample. Data expressed as Mean ± SEM. Two-way ANOVA with Tukey’s multiple comparisons test was done. *P < 0.05, Cell lysates was collected from three different passages with WB analysis done on each passage. The original full-length gels (and the replicates) are shown in Supplementary Figure S3, where the gel sections provided in this image are highlighted in a red box.

It is well-known that following exposure to oxidative stress, cells express and/or upregulate a range of different antioxidant proteins, including calatase, SOD, and thioridoxins (Trx) in oder to combat cellular damage (Chaudhary et al., 2023; Forman and Zhang, 2021; Garg et al., 2020; Godic et al., 2014). As such, we screened a panel of antioxidant proteins via Western blot analysis, to determine changes in their protein expression levels. Both siRNA knockdown and rCLIC treated cells were exposed to 30 min of UV irradiation (+UV), followed by collection of whole cell lysates. Western blot analysis was then performed using specific antibodies against CLIC1, CLIC4, catalase, SOD1, Trx and nitrotyrosine, along with β-actin used as the loading control. 10μg of whole cell lysate protein was loaded into the gel and samples not exposed to UV (No UV) were used as the controls, as shown in Figure 5. Samples were assessed for nitrotyrosine expression, a marker of oxidative stress and inflammation, to further validate the effect of siRNA knockdowns and rCLICs treatment of HDF cells. The original full-length gels and the replicates are shown in Supplementary Figure S3.

As seen in Figure 5, knockdown of CLIC1 or both CLIC1 and CLIC4 proteins (DKD), but not knockdown of CLIC4 alone, resulted in increased levels of nitrotyrosine expression, indicating that the cells were experiencing some level of oxidative stress, irrespective of UV irradiation, following the loss of CLIC1 or possibly both CLIC1 and CLIC4 proteins. On the other hand, rCLIC1 or rCLIC4 treatment resulted in a significant decrease in nitrotyrosine levels following UV exposure, while no change in nitrotyrosine expression was observed in the absence of UV. These findings strongly suggest that treatment of HDF cells with rCLIC1 or rCLIC4 reduces UV-induced oxidative stress, while down-regulating CLIC expression increases cellular oxidative load. Western blot analysis of the other three antioxidant marker proteins in the absence of UV exposure, also validated this, as the DKD knockdown cells showed significant increase in the levels of all three oxidative stress related antioxidant proteins (catalase, SOD1 and Trx). In the absence of UV irradiation, CLIC1-KD cells also showed significant increase in the expression levels of SOD1 and though Trx levels were higher than the control cells (HDF and Scmb C) it was not deemed significant. While, down-regulating CLIC4 expression in CLIC4-KD cells showed a moderate increase in SOD1 expression and slight increase in Trx levels, but they were not significantly different to that of the control cells. Furthermore, treatment with either rCLIC1 or rCLIC4 did not alter or affect the expression levels of any of the oxidative stress related proteins in the HDF cells thereby, strongly suggesting that rCLICs treatment have little to no adverse effect on the cells. On the other hand, exposure to UV irradiation resulted in HDF and Scmb Control cells, both showing increased expression levels of the antioxidant proteins (catalase, SOD1 and Trx) as expected. Comparison of the expression levels between the siRNA knockdown and rCLIC treated cells, with the Control cells (HDF and Scmb C) in the presence of UV (+UV) resulted in all the knockdown cells showing significantly increased levels of catalase. Interestingly, rCLIC1 treated cells resulted in signifiacntly lower levels of Trx expression.

A recent study by Al Khamici H et al. (2022) showed that depleting CLIC4 expression in 6DT1 breast tumor cells led to increased ROS levels, mitochondrial hyperactivity, and heightened sensitivity to H_2_O_2_-induced apoptosis, partly due to reduced expression of Bcl2 and UCP2 (Al Khamici et al., 2022). Another study by Suh KS et al. (2007) showed that knockdown of CLIC4 protein by antisense or shRNA prevents Ca^2+−^induced keratin 1, keratin 10 and filaggrin expression and causes cell cycle arrest in both human and murine keratinocytes undergoing differentiation (Suh et al., 2007). On the other hand, several studies have implicated CLIC1 as a potential sensor of cell oxidation (Averaimo et al., 2010). Suppression of CLIC1 expression through gene knocked-out (CLIC1^−/−^) or using the specific inhibitor IAA94 in human umbilical vein endothelial cells (HUVECs) reduced ROS production, increased SOD enzyme activity, and significantly decreased MDA level (Xu et al., 2016). HUVECs cells when treated with H_2_O_2_ induced endothelial oxidative damage and showed enhanced CLIC1 expression (Xu et al., 2016). There are now also several studies that show enhanced CLIC1 expression in a number of disease states, that involve oxidative stress, including tumors. A study by Lee et al. (2019) showed that in A549 lung cancer cells, CLIC1 knockdown led to elevated basal ROS and intracellular Ca^2+^ levels and the cells showed increased DNA damage, as well as, enhanced activation of the JNK cellular pathway (Lee et al., 2019). Depletion of CLIC1 using siRNA in human oesophageal squamous cell carcinoma, also induced apoptosis via the TLR2/JNK pathway (Kobayashi et al., 2018). Studies by Ponnalagu et al., 2018 and 2022, highlighted the involvement of CLIC4 and CLIC5 in maintaining calcium homeostasis and mitochondrial ROS generation and aiding in cardio protection from *in vivo* ischemia-reperfusion (Ponnalagu et al., 2022; Ponnalagu et al., 2018).

Growing evidence also suggests potential roles of CLIC-like proteins in redox regulation. In invertebrates, DmCLIC regulates oxidative stress signaling in *Drosophila*, with its loss leading to increased expression of redox-related proteins and altered ethanol sensitivity (Weston et al., 2021). Our current study also showed increased expression of some of the redox related proteins following CLIC1 or CLIC4 depletion in HDF cells (Figure 5). It would be interesting to conduct comparable studies in the future in order to confirm the general nature of these findings in keratinocyte cells and also across different cell types but was beyond the scope of this study. However, based on the results shown in Figures 4, 5, this increased expression of redox-related proteins might also account for the increase in the oxidoreductase activity seen for the CLIC knockdown cells in the HEDS assay (Figure 4) following UV exposure. This increase in expression of redox-related proteins may also contribute to protecting the cells from UV-induced damage, as a result the UV-treated knockdown cells did not show greater cell damage, nor increased ROS levels, in comparison to the UV-treated control cells (Figure 2) and warrants further investigation. Taking into consideration the widespread cellular expression and subcellular localisation of individual CLIC proteins (Ulmasov et al., 2007), the co-expression of multiple CLICs in different cell and tissue types, together with their high degree of evolutionary conservation (Littler et al., 2008; Elter et al., 2007; Gururaja et al., 2017), it is now evident that several different cellular processes are associated with the redox regulatory roles of the different CLICs, and this may also depend on their cellular localisation and tissue type.

### 3.6 Exogenously added rCLIC1 or rCLIC4 protein rescues and protects siRNA knockdown HDF cells from UV exposed cellular damage

**FIGURE 6:**
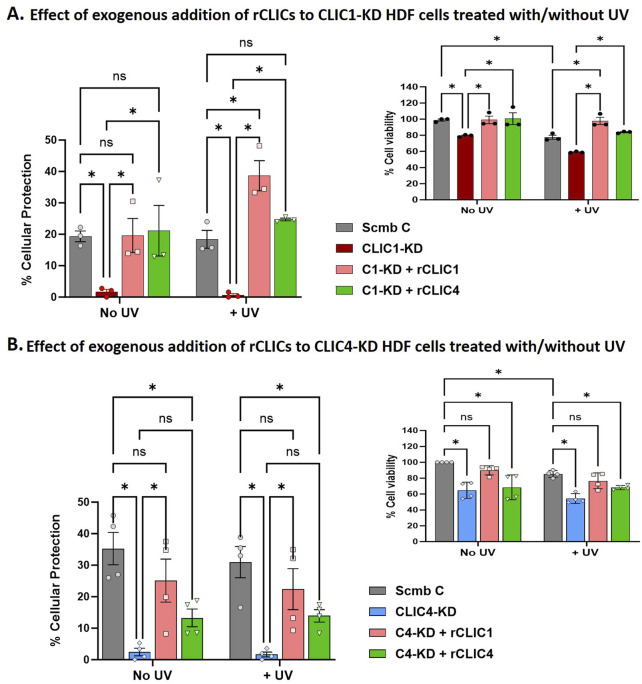
Percentage cellular viability and the corresponding percentage cellular protection of siRNA knockdown HDF cells treated with different rCLICs prior to them being subjected to UV irradiation. Percentage Cell Viability and Cellular protection of either CLIC1 knockdown (CLIC1-KD, **(A)** or CLIC4 knockdown (CLIC4-KD, **(B)** treated with rCLIC1 (pink), or rCLIC4 (green) following no treatment (No UV) or exposed to 30 min UV (+UV). Cells treated with scramble siRNA and not rCLIC proteins were used as Control (Scmb C, silver) and compared to cells treated with CLIC1 siRNA (CLIC1-KD, red) or CLIC4 siRNA (CLIC4-KD, blue) but not exogenous proteins. Two-way ANOVA with Tukey’s multiple comparisons test was done. Data represented as Mean ± SEM. Data was collected from three different passages with each passage run in triplicate. *p < 0.05, ns = non-significant.

We next investigated the effect of exogenously adding rCLIC1 or rCLIC4 to CLIC1-KD or CLIC4-KD HDF cells prior to UV exposure. Cells treated with scrambled siRNA (Scmb C), CLIC1-KD and CLIC4-KD cells, that were not exposed to UV treatment, were used as the controls. Figure 6 shows the percentage cell viability and the corresponding percentage cellular protection of the CLIC1-KD/CLIC4-KD siRNA knockdown cells treated with/without rCLIC proteins in the absence or presence of UV irradiation.

Figure 6 shows significant reduction in cell viability in CLIC1-KD and CLIC4-KD cells in the absence or presence of UV irradiation in comparison to Scmb Control cells. As was also previously seen in Figure 2, it is clear that downregulation of CLIC1 or CLIC4 expression in skin cells (HDF and HKE) causes loss of cell viability, increased basal ROS levels, and increases their susceptibility to oxidative damage. Interestingly, CLIC1-KD cells treated with exogenous rCLIC1 (C1-KD + rCLIC1) or rCLIC4 (C1-KD + rCLIC4), showed greater percentage cell viability in comparison to the non-treated CLIC1-KD cells, both in the absence and presence of oxidative damage. Treatment of CLIC1-KD cells with exogenously added rCLICs, showed restored cell viability, with no significant difference compared to the Control cells lines expressing endogenous levels of CLIC proteins. Following UV exposure, CLIC1-KD cells treated with rCLIC1 showed greater cell viability and the greatest amount of protection in comparison to the control cells and rCLIC4 treated knockdown cells. These findings provide further evidence that addition of exogenous rCLIC1 or rCLIC4 protect cells resulting in increased cell viability. On the other hand, CLIC4-KD cells in the absence or presence of UV irradiation, showed significant increase in cellular protection only when treated with rCLIC1 (C4-KD + rCLIC1), restoring them to viability levels equal to that of the Control cells; while treatment with rCLIC4, although resulted in an observable increase in cell viability, did not reach a level of significance (p < 0.05). This result points to a potential difference in the intracellular actions of these two proteins, CLIC1 and CLIC4, including their specific roles in the antioxidant processes, interactions in signaling cascades and intracellular localisation that warrants further investigation.

## 4 Conclusion

This study has demonstrated for the first time the cellular antioxidant capabilities of recombinant CLIC proteins specifically against UVC-induced damage in human skin cells. Exogenous addition of either rCLIC1 or rCLIC4 not only provided cellular protection against UVC-induce damage in fibroblast and keratinocyte cells in culture but upon treatment, were also able to protect the CLIC knockdown cells against UV damage and restore cell viability. Future studies to understand whether these proteins are also effective in protecting cells against UV-A and UV-B are currently being planned. We have also demonstrated rCLIC proteins regulate cellular ROS levels via their oxidoreductase activity and they provide similar levels of protection against UV-damage to that of Grx and GST-Ω proteins. In this study, we have also shown changes in the expression pattern of redox-related proteins following CLIC1 and CLIC4 knockdowns, whereas, little to no changes were seen following rCLIC treatment, both in the presence and absence of UV irradiation. CLIC1 or CLIC4 and double knockdown cells all showed enhanced levels of catalase, SOD1 and thioredoxin expression, as well as increased expression of the oxidative stress marker protein nitrotyrosine. Taking into consideration our previous cell study (Hossain et al., 2025) along with the findings from the current study, there is now overwhelming evidence that members of the CLIC family play a critical regulatory role in cellular redox processes and in cellular protection against oxidative stress. It is also reasonable to include these proteins as members of the natural human antioxidant defence system, which includes enzymes such as superoxide dismutase (SOD), catalase (CAT), and glutathione peroxidase (GPx), which are critical for maintenance of the cell’s overall health and viability.

## Data Availability

The original contributions presented in the study are included in the article/Supplementary Material, further inquiries can be directed to the corresponding author.
