# Supplementary material for: CLIC1 and CLIC4 demonstrate cell protective antioxidant activity against UV exposure
Source: Front Cell Dev Biol. 2025 Nov 18;13:1674374. doi: 10.3389/fcell.2025.1674374 (PMC12669148; doi:10.3389/fcell.2025.1674374)
Supplement: Supplementary file 1 [file DataSheet1.pdf]

Supplementary Material

CLIC1 and CLIC4 Demonstrate Cell Protective Antioxidant Activity Against UV Exposure

KR Hossain[1#], A Alghalayini[1], DR Turkewitz[1] and SM Valenzuela[1*#].
School of Life Sciences, University of Technology Sydney, Sydney, NSW 2007, Australia; khondker.hossain@uts.edu.au (K.R.H.); amani.alghalayini@uts.edu.au (A.A.); daniel.r.turkewitz@alumni.uts.edu.au (D.R.T.);
#Correspondence: stella.valenzuela@uts.edu.au (S.M.V); Tel.: +61-2-95141917

1 Supplementary Data

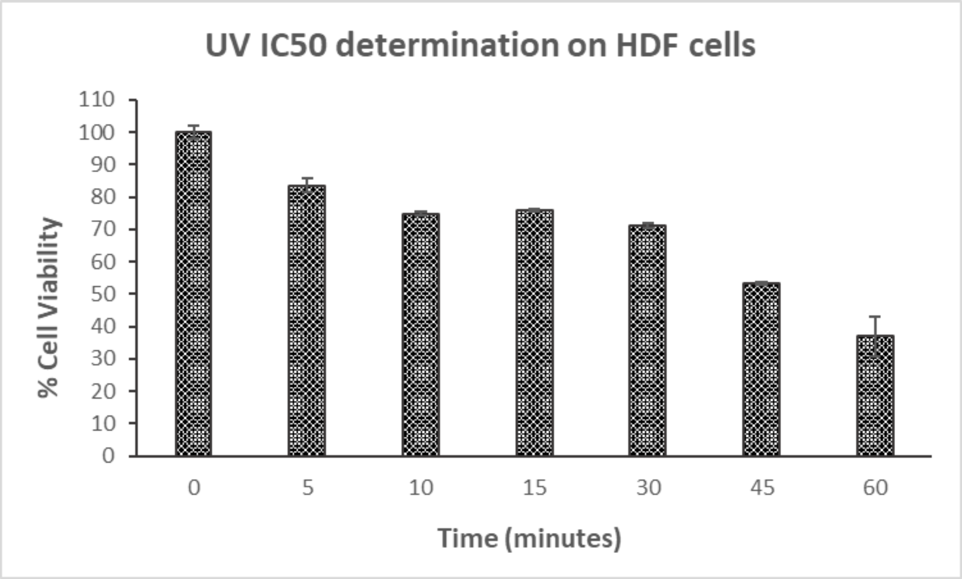

Supplementary Figure S1: The Half-maximal inhibitory concentration (IC_{50}) of UV exposure on HDF cells shows a gradual decrease in cell viability following longer exposure time.

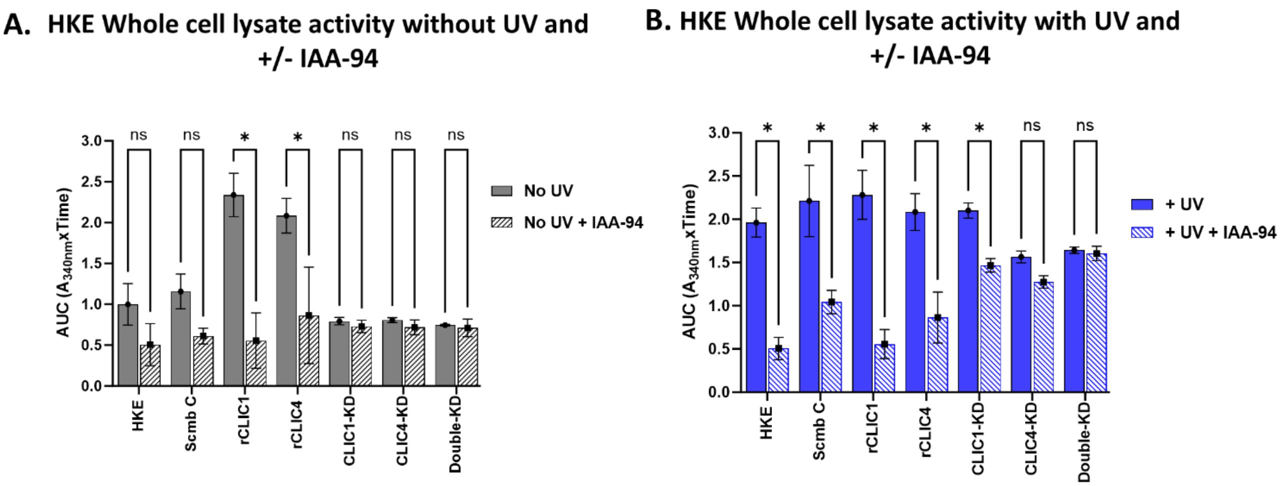

Supplementary Figure S2: Area Under the Curve (AUC) showing Oxidoreductase Activity of whole cell lysates collected from rCLIC treated or siRNA knockdown HKE cells treated without(A) and with UV (B) in the presence or absence of drug IAA-94. Cell lysates collected from HKE cells following no treatment with UV (**A**) or exposed to UV (**B**) and then pre-incubated without or with IAA-94 are represented as solid or stripped bars respectively. Two-way ANOVA with Tukey's multiple comparisons test was done. *$P<0.05$, ns= not significant. Data represented as Mean ± SEM. For each experiment, data was collected from 3 different passages with each passage run in triplicate.

A. Anti-body Mixture: SOD/Catalase/Nitrotyrosine/B-actin

rCLIC Treated Cells

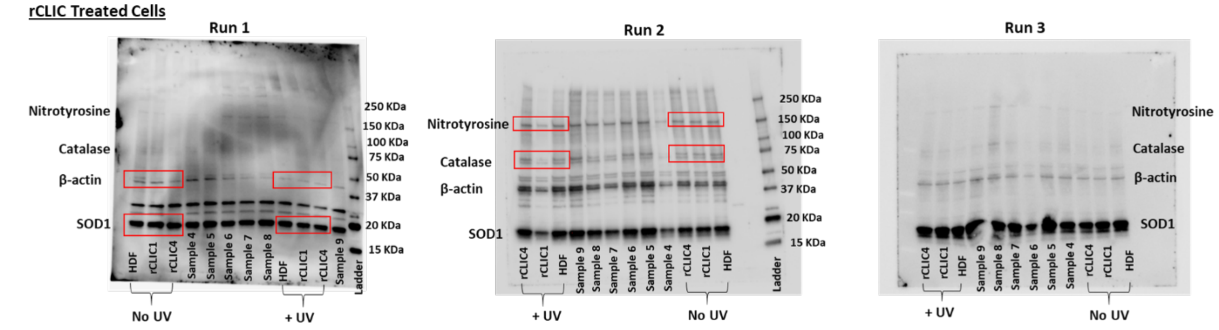

siRNA Knockdown Cells

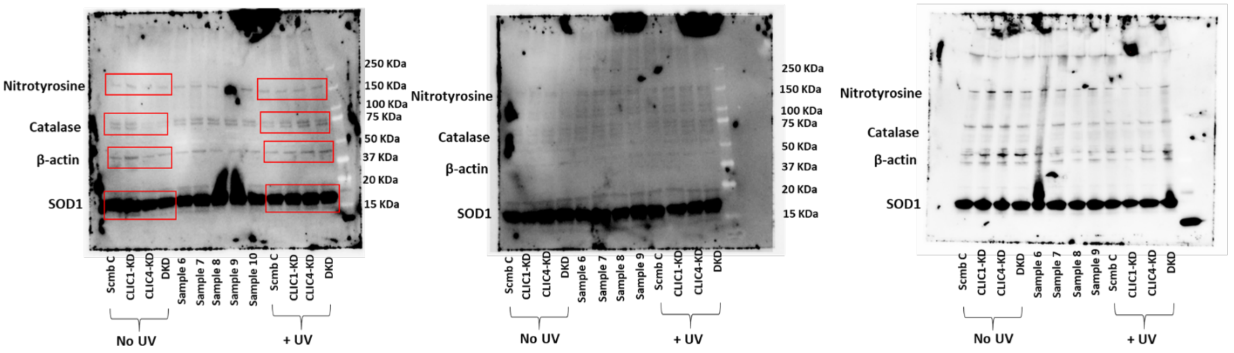

B. Anti-body: Trx

rCLIC Treated Cells

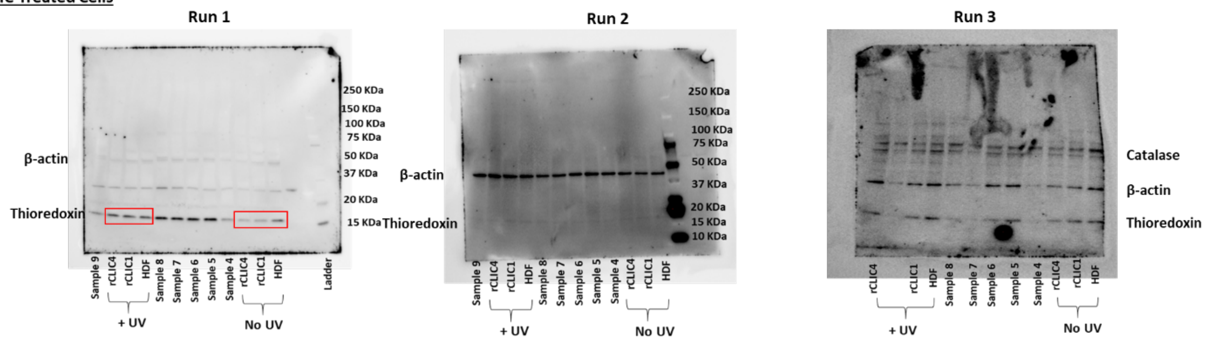

siRNA Knockdown Cells

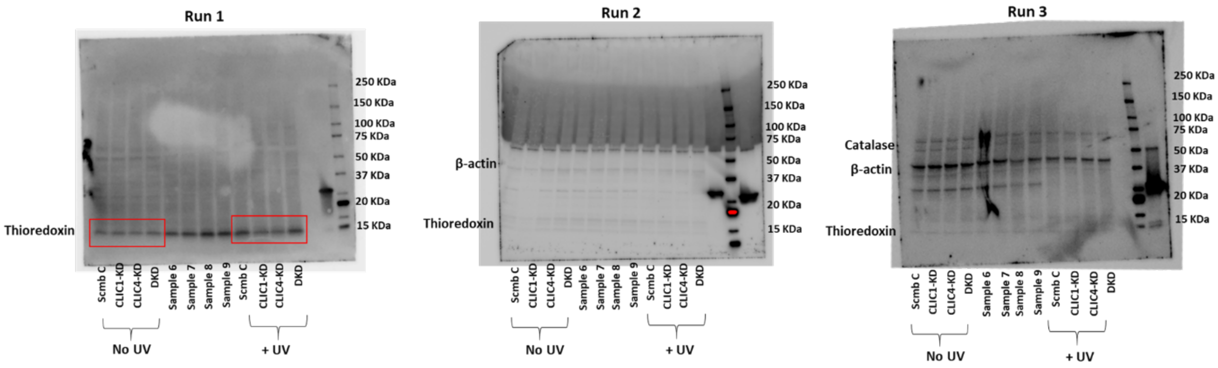

C. Anti-body: CLIC1

rCLIC Treated Cells

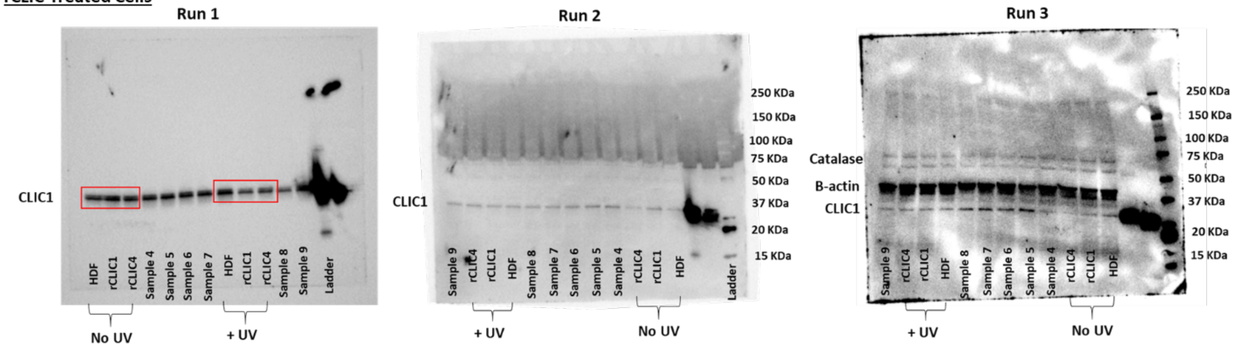

siRNA Knockdown Cells

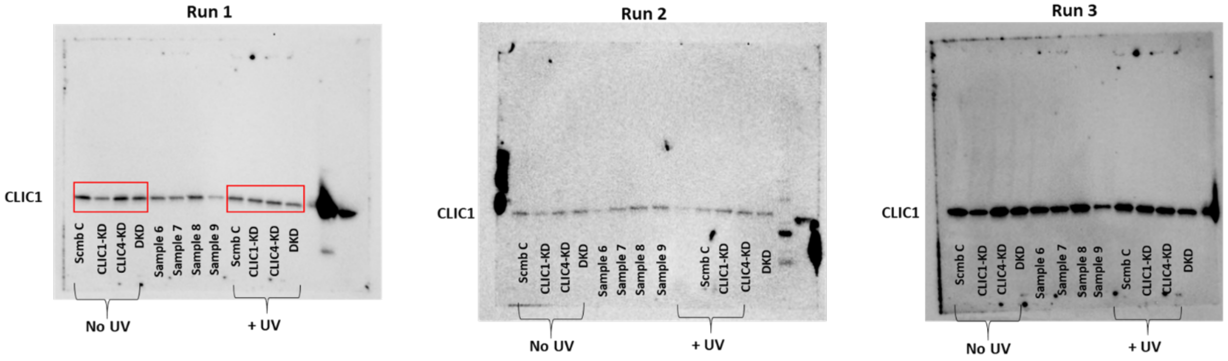

D. Anti-body: CLIC4

rCLIC Treated Cells

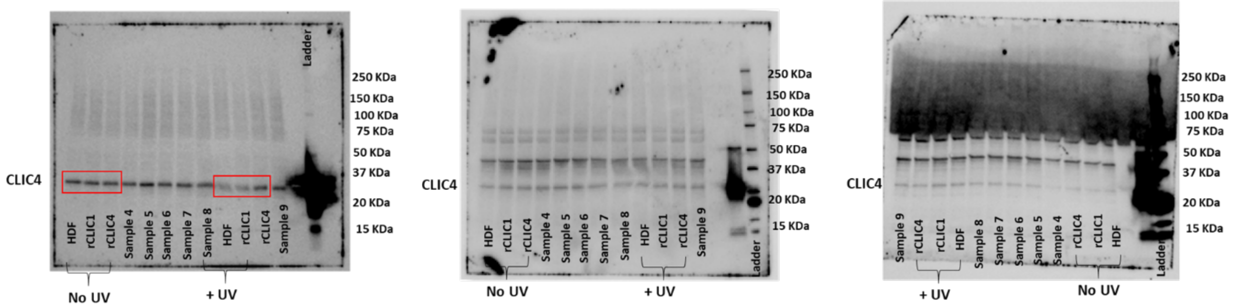

siRNA Knockdown Cells

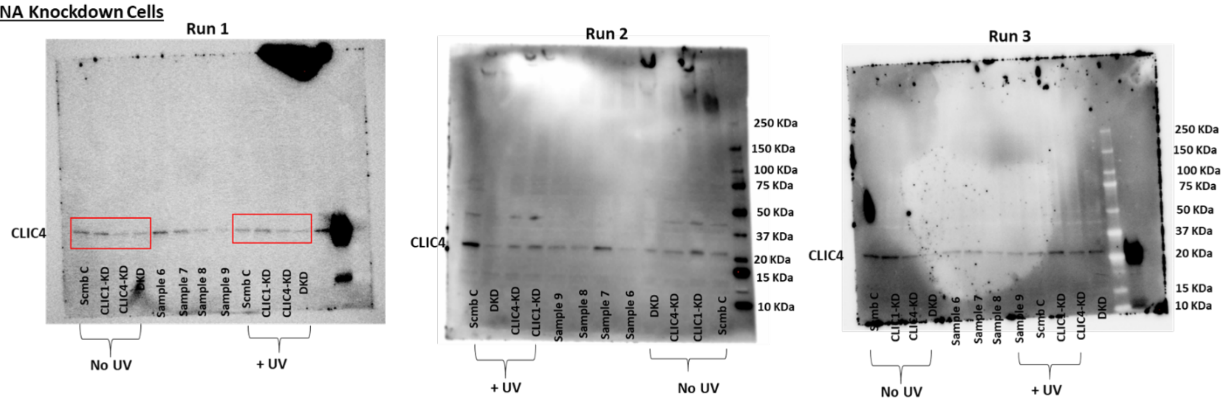

Supplementary Figure S3: The original full-length gels and the replicates for the western blot

analysis shown in Figure 5, with the gel sections provided in Figure 5[i] highlighted in a red box. A) Original and replicate gels (n =3) stained with antibody cocktail containing Anti-Nitrotyrosine, Anti-Catalase, Anti-β-actin and Anti-SOD1. **B)** Original and replicate gels (n =3) stained with Anti-Thioredoxin antibody. **C)** Original and replicate gels (n =3) stained with Anti-CLIC1 antibody. **D)** Original and replicate gels (n =3) stained with Anti-CLIC4 antibody. Western blot analysis of Whole cell lysates collected from three different passage of HDF cells treated with either rCLIC or siRNA knockdown following no UV treatment (No UV) and UV treatment (+ UV) for each of the antibodies are shown in the bottom panel in each gel respectively. 10 µg of protein was loaded for each sample and densitometry analysis was conducted in Fiji/ImageJ and further processed in GraphPad Prism 8 (GraphPad Software, La Jolla California USA). The data is shown in the manuscript in Figure 5 ii-vii for the different antibodies.
